# The Efficacy of Vildagliptin Concomitant With Insulin Therapy in Type 2 Diabetic Subjects

**DOI:** 10.14740/jocmr2057w

**Published:** 2015-03-01

**Authors:** Daisuke Ito, Kazuyuki Inoue, Kimie Kaneko, Morifumi Yanagisawa, Takashi Sumita, Yuichi Ikegami, Takuya Awata, Hitoshi Ishida, Shigehiro Katayama, Kouichi Inukai

**Affiliations:** a Division of Endocrinology and Diabetes, Saitama Medical University, 38, Morohongo, Moroyama, Iruma-gun, Saitama 350-0495, Japan; b Division of Internal Medicine, Ogawa Red Cross Hospital, 1525, Ogawa, Ogawa-machi, Hiki, Saitama 355-0397, Japan; c Satsuki Medical Clinic, 1-471, Ogawa, Ogawa-machi, Hiki, Saitama 355-0397, Japan; d Third Department of Internal Medicine, Division of Diabetes, Endocrinology and Metabolism, Kyorin University School of Medicine, 6-20-2, Shinkawa, Mitaka, Tokyo, Japan

**Keywords:** Type 2 diabetes, Insulin therapy, Vildagliptin, Sitagliptin

## Abstract

**Background:**

In Japan, dipeptidyl peptidase 4 (DPP4) inhibitors have become standard therapeutic agents for type 2 diabetes, and numbers of patients receiving insulin therapy combined with DPP4 inhibitors, which is a highly effective regimen, are increasing.

**Methods:**

In this study, we evaluated the efficacy of vildagliptin administered at the dose of 100 mg twice daily in 57 patients with type 2 diabetes already receiving insulin treatment.

**Results:**

The 36 patients who simply received add-on vildagliptin showed a 0.6% decrease in HbA1c levels, despite a marked insulin dose reduction, mainly bolus insulin, of approximately 8.3 units. In addition, body mass index exhibited a significant negative correlation with the efficacy of vildagliptin, i.e., ΔHbA1c. On the other hand, the 21 patients switched from 50 mg of sitagliptin to vildagliptin showed HbA1c decreases approaching 0.7%.

**Conclusion:**

Taking into consideration that twice-daily oral vildagliptin has already been reported to be advantageous in reducing postprandial hyperglycemia, this drug was suggested to be more effective in reducing HbA1c than sitagliptin under conditions in which it is used as a supplement to basal insulin, as in this study.

## Introduction

Dipeptidyl peptidase 4 (DPP4) inhibitors increase the activity of glucagon-like peptide-1 (GLP-1) and gastric inhibitory polypeptide (GIP), which are incretin hormones secreted by gastrointestinal mucosal cells in response to carbohydrate intake, thereby promoting glucose-responsive insulin secretion [1]. Accordingly, because these drugs reduce both fasting and postprandial blood glucose levels with no risk of hypoglycemia, they clearly have higher efficacy than sulfonylurea agents, which are conventional insulin secretagogues [2]. Beyond glycemic control, DPP4 inhibitors were reported to have pleiotropic effects such as body weight loss [3] and improvement of lipid profiles [4]. It is estimated that DPP4 inhibitors have been used in as many as 3 million patients with type 2 diabetes since sitagliptin was first launched on the Japanese market in 2010. These drugs seem to be very highly effective in Japanese diabetic patients, who mainly have impaired insulin secretion. At present, seven DPP4 inhibitors, i.e. sitagliptin, alogliptin, vildagliptin, linagliptin, teneligliptin, anagliptin and saxagliptin, are available in Japan [5-12]. Among these, vildagliptin is considered to have a superior postprandial hypoglycemic effect, because it covalently binds to DPP4 [13], thereby markedly increasing postprandial GLP-1 activity, although it has to be administered orally twice daily due to its relatively short half-life [14].

Combined DPP4 inhibitor and insulin therapy was introduced in Japan in 2013, and numerous patients with type 2 diabetes receiving insulin began to additionally receive DPP4 inhibitors. However, to date, few studies have examined the efficacy of vildagliptin administered in combination with insulin therapy in Japanese patients. Therefore, in this study, we determined the efficacy of vildagliptin administered in combination with insulin. We also conducted a test in which sitagliptin, which is currently the most widely used DPP4 inhibitor in Japan, was switched to vildagliptin to compare the glucose-lowering effects of these two agents.

## Materials and Methods

### Subjects

**Figure 1:**
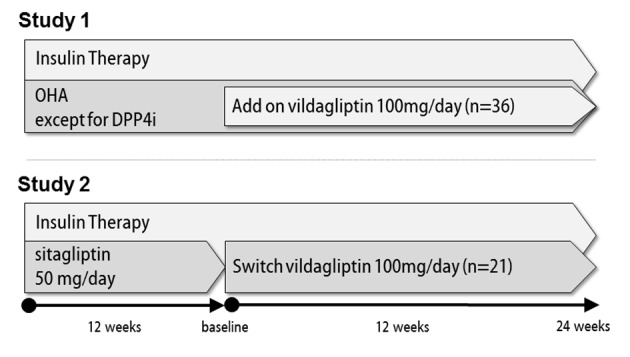
Outlines of studies 1 and 2.

This was a multicenter, open-label, prospective, observational clinical trial. After obtaining approval from the Ethics Review Board of the Japanese Red Cross Ogawa Hospital, which played a central role in this study, 57 patients from multiple institutions with type 2 diabetes, all of whom were receiving insulin, were enrolled as study participants. The study design is outlined in Figure 1. In study 1, 36 patients not receiving an oral DPP4 inhibitor were given 100 mg of add-on vildagliptin orally twice daily throughout the study period of 24 weeks. The insulin dose was reduced as appropriate at the start of the study and also changed as appropriate according to glycemic control status during the observation period at the discretion of the attending physician. In study 2, 21 patients receiving 50 mg of oral sitagliptin once daily were switched to 100 mg of vildagliptin orally twice daily and then observed for 24 weeks. The insulin dose was not changed at the time of switching DPP4 inhibitor treatment, but was changed as appropriate according to glycemic control status during the observation period. When we calculated the mean bolus insulin dose, we evaluated the bolus insulin dosage of patients treated with only basal insulin, as 0 units. In both study 1 and study 2, other oral hypoglycemic agents were left unchanged during the study period, and patients with severe hypoglycemia and those in whom HbA1c was maintained at 9.0% for 3 months or longer were withdrawn from the study. All patients gave written consent before participating in this study.

### Outcome measures

The serum HbA1c levels, 2-h postprandial plasma glucose levels after breakfast, plasma C-peptide levels, body weight, and presence/absence of adverse events such as hypoglycemia were routinely monitored during the study period. These parameters were measured employing routine laboratory techniques. Exclusion criteria at enrollment included liver dysfunction (aspartate aminotransferase (AST)/alanine aminotransferase (ALT) > 50 IU/L), renal dysfunction (creatinine > 1.5 mg/dL) and severe obesity (body mass index (BMI) > 35 kg/m^2^).

### Statistical analysis

Data are shown as means ± standard error (SE). P < 0.05 by paired *t* test was considered to denote a statistically significant difference. STATA SE 11 was used for all statistical analyses.

## Results

**Figure 2:**
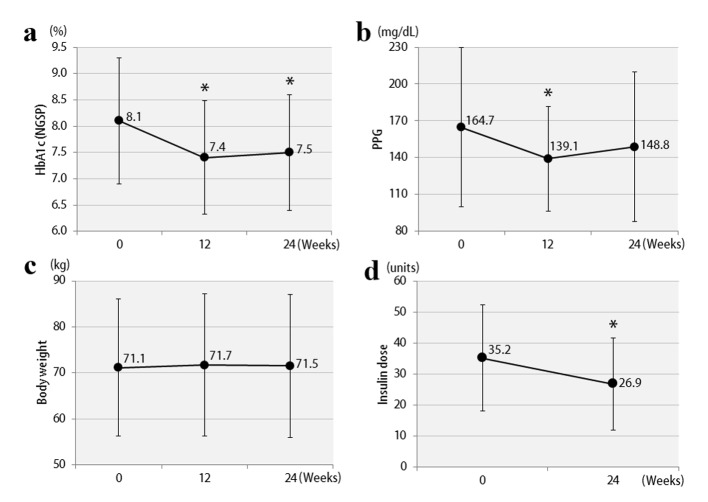
Changes in clinical parameters after 12 and 24 weeks in study 1: (a) HbA1c; (b) 2-h postprandial plasma glucose; (c) body weights; (d) insulin doses. *P < 0.05 compared with the baseline.

**Figure 3:**
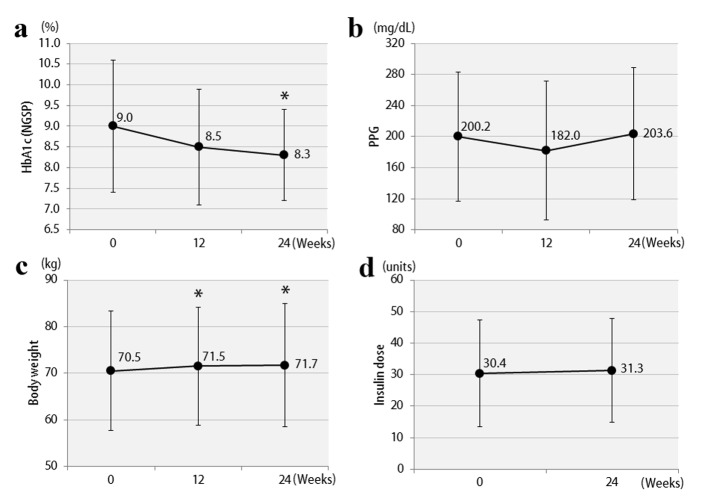
The changes in clinical parameters after 12 and 24 weeks in study 2: (a) HbA1c; (b) 2-h postprandial plasma glucose; (c) body weights; (d) insulin doses. *P < 0.05 compared with the baseline.

**Figure 4:**
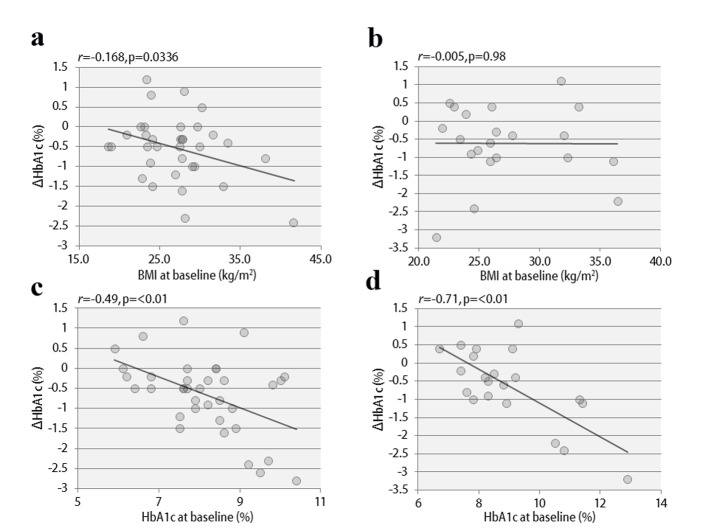
The correlations between ΔHbA1c and BMI at baseline in study 1 (a) and study 2 (b), and between ΔHbA1c and HbA1c at baseline in study 1 (c) and study 2 (d).

The clinical characteristics of the 57 enrolled patients at the start of the study are shown, separately for studies 1 and 2, in Table 1. The patients were being treated with insulin, and the duration of diabetes was at least 10 years. In addition, there was a tendency for obesity, with the mean BMI exceeding 25. Therefore, the mean total insulin dose was ≥ 30 units daily, which is considered to be high for Japanese patients. The study involved poorly controlled patients with a mean HbA1c level ≥ 8%. The results of study 1 are shown in Figure 2. In the vildagliptin add-on therapy group, the mean HbA1c level decreased from 8.1% to 7.5% (Fig. 2a) and, notably, the mean daily insulin dose could be reduced significantly, by 8.3 units (Fig. 2d). The mean dose of rapid acting insulin, i.e. bolus insulin, was reduced by 7.8 units (from 26.4 to 18.6 units), while that of basal insulin was reduced by 0.5 units (from 8.8 to 8.3 units). Thus, bolus insulin injected before meals accounted for most of the insulin dose reduction. The 2-h postprandial plasma glucose levels also decreased significantly (Fig. 2b). There were no significant changes in body weight (Fig. 2c) or 2-h postprandial plasma C-peptide levels. The results of study 2 are shown in Figure 3. After switching from sitagliptin to vildagliptin, the mean HbA1c level improved by 0.7%, down from 9.0%, despite almost no change in the insulin dose (Fig. 3a, d). The 2-h postprandial plasma glucose levels did not change (Fig. 3b), suggesting that mainly improvement and stabilization of fasting blood glucose levels had contributed to the significant improvement in HbA1c levels. Mean body weight increased significantly by 1.2 kg (Fig. 3c), but there was no significant change in 2-h plasma postprandial C-peptide levels. Figure 4 presents the relationships between the efficacy of vildagliptin, i.e., ΔHbA1c, and BMI and baseline HbA1c. BMI exhibited a significant negative correlation with ΔHbA1c in study 1, while no relationships were observed in study 2. Baseline HbA1c was strongly associated with ΔHbA1c in both studies. None of the patients were withdrawn from either study as there were no marked deteriorations of blood glucose levels. In study 1, two patients developed mild hypoglycemia, but there were no episodes of severe hypoglycemia. In addition, no safety problems were seen in any of the 57 patients.

**Table 1:** Characteristics of the Patients in Each Study Group

	Study 1	Study 2
Number of subjects	36	21
Age (years)	64.1 ± 11.3	57.5 ± 12.5
Sex male/female (%)	63.9/36.1	47.6/52.4
Durations for diabetes (years)	14.4 ± 6.7	13.4 ± 4.7
Body weight (kg)	71.1 ± 14.9	70.5 ± 12.8
BMI (kg/m^2^)	26.8 ± 5.1	27.1 ± 4.6
HbA1c (NGSP) (%)	8.1 ± 1.2	9.0 ± 1.6
2-h postprandial plasma glucose (mg/dL)	164.7 ± 64.7	200.2 ± 83.2
2-h postprandial CPR (ng/mL)	2.5 ± 2.3	1.9 ± 1.7
Insulin total doses (units)	35.2 ± 17.1	30.4 ± 17.0

## Discussion

DPP4 inhibitors are therapeutic agents that, in combination with insulin treatment, can be expected to further improve blood glucose control [15]. In particular, add-on DPP4 inhibitors in patients receiving treatment with basal insulin alone, i.e., basal supported oral therapy (BOT), are reportedly more effective than premixed insulin preparations or intensive insulin therapy [15]. These data suggest that the postprandial hypoglycemic effect of DPP4 inhibitors and the fasting hypoglycemic effect of basal insulin make this the most appropriate combination. On the other hand, if DPP4 inhibitors are combined with premixed insulin preparations or bolus insulin, it is necessary to adjust the insulin dose, particularly that of bolus insulin, to avoid hypoglycemia. In the present study as well, the bolus insulin dose was reduced by 7.8 units, while the basal insulin dose was only slightly reduced (by 0.5 units). Add-on vildagliptin allowed some patients to finally be switched from basal-bolus insulin therapy to BOT. These results demonstrate that the addition of DPP4 inhibitors to insulin therapy allows substantial reductions in bolus insulin doses.

Considering the results of previous meta-analyses and other studies, vildagliptin is likely to have the strongest hypoglycemic effect among the many DPP4 inhibitors currently available [16]. As vildagliptin covalently binds to DPP4 [13] and thereby markedly increases postprandialGLP-1 activity, it is considered to have a particularly excellent postprandial hypoglycemic effect [14]. In addition, it has been reported that, as a secondary effect of markedly reducing postprandial blood glucose levels, vildagliptin has a more pronounced effect in lowering blood oxidative stress and inflammatory markers than other DPP4 inhibitors [17]. As the reductions in these substances are likely to improve insulin resistance, this specific effect of vildagliptin depends on the degree of obesity (as reflected by high BMI). In fact, vildagliptin was previously reported to be more effective in diabetic patients with a tendency towards obesity [18]. In the present study, BMI was also negatively associated with ΔHbA1c, an observation consistent with this hypothesis. This is in marked contrast to the data on sitagliptin, the effect of which is reported to be attenuated at BMI values exceeding 25 [19]. Therefore, the insulin-resistance-improving effect of vildagliptin apparently enhances the fasting hypoglycemic effect [16] and, furthermore, produces greater reductions in HbA1c levels than other DPP4 inhibitors [20]. Although the usual dose of sitagliptin is 100 mg daily in countries other than Japan, 50 mg daily is adopted as the routine dose in Japan, because a phase III clinical trial in Japanese subjects showed no difference in hypoglycemic effects between 50 and 100 mg of sitagliptin [21, 22]. In the present study, the treatment switch from 50 mg of sitagliptin to vildagliptin resulted in a marked improvement in HbA1c levels. This result suggests that insulin therapy plus vildagliptin, with its marked postprandial hypoglycemic effect, is a very beneficial combination, although the patient group with a relatively high BMI appeared to benefit more than leaner subjects in the present study.

In conclusion, we were able to reduce the bolus insulin dose and further improve postprandial blood glucose and HbA1c levels by adding vildagliptin to the treatment regimens of patients with type 2 diabetes receiving insulin. Furthermore, we also demonstrated the usefulness of switching from sitagliptin to vildagliptin. The results of this study are considered to be clinically significant in that they present a new effective means of achieving glycemic control in type 2 diabetic patients with insufficient glycemic control while treated with insulin alone.

## References

[1] Tahrani AA, Bailey CJ, Del Prato S, Barnett AH (2011). Management of type 2 diabetes: new and future developments in treatment. Lancet.

[2] Arai K, Matoba K, Hirao K, Matsuba I, Takai M, Takeda H, Kanamori A (2010). Present status of sulfonylurea treatment for type 2 diabetes in Japan: second report of a cross-sectional survey of 15,652 patients. Endocr J.

[3] Shigematsu E, Yamakawa T, Kadonosono K, Terauchi Y (2014). Effect of sitagliptin on lipid profile in patients with type 2 diabetes mellitus. J Clin Med Res.

[4] Yanai H, Adachi H, Hamasaki H, Masui Y, Yoshikawa R, Moriyama S, Mishima S (2012). Effects of 6-month sitagliptin treatment on glucose and lipid metabolism, blood pressure, body weight and renal function in type 2 diabetic patients: a chart-based analysis. J Clin Med Res.

[5] Schweizer A, Dejager S, Foley JE, Kothny W (2011). Assessing the general safety and tolerability of vildagliptin: value of pooled analyses from a large safety database versus evaluation of individual studies. Vasc Health Risk Manag.

[6] Pratley RE (2009). Alogliptin: a new, highly selective dipeptidyl peptidase-4 inhibitor for the treatment of type 2 diabetes. Expert Opin Pharmacother.

[7] Araki E, Kawamori R, Inagaki N, Watada H, Hayashi N, Horie Y, Sarashina A (2013). Long-term safety of linagliptin monotherapy in Japanese patients with type 2 diabetes. Diabetes Obes Metab.

[8] Kutoh E, Hirate M, Ikeno Y (2014). Teneligliptin as an initial therapy for newly diagnosed, drug naive subjects with type 2 diabetes. J Clin Med Res.

[9] Ervinna N, Mita T, Yasunari E, Azuma K, Tanaka R, Fujimura S, Sukmawati D (2013). Anagliptin, a DPP-4 inhibitor, suppresses proliferation of vascular smooth muscles and monocyte inflammatory reaction and attenuates atherosclerosis in male apo E-deficient mice. Endocrinology.

[10] Gerrald KR, Van Scoyoc E, Wines RC, Runge T, Jonas DE (2012). Saxagliptin and sitagliptin in adult patients with type 2 diabetes: a systematic review and meta-analysis. Diabetes Obes Metab.

[11] Kanamori A, Matsuba I (2013). Factors associated with reduced efficacy of sitagliptin therapy: analysis of 93 patients with type 2 diabetes treated for 1.5 years or longer. J Clin Med Res.

[12] Kubota A, Maeda H, Kanamori A, Matoba K, Jin Y, Minagawa F, Obana M (2012). Pleiotropic effects of sitagliptin in the treatment of type 2 diabetes mellitus patients. J Clin Med Res.

[13] Zettl H, Schubert-Zsilavecz M, Steinhilber D (2010). Medicinal Chemistry of Incretin Mimetics and DPP-4 Inhibitors. ChemMedChem.

[14] Sakamoto M, Nishimura R, Irako T, Tsujino D, Ando K, Utsunomiya K (2012). Comparison of vildagliptin twice daily vs. sitagliptin once daily using continuous glucose monitoring (CGM): crossover pilot study (J-VICTORIA study). Cardiovasc Diabetol.

[15] Katsuno T, Ikeda H, Ida K, Miyagawa J, Namba M (2013). Add-on therapy with the DPP-4 inhibitor sitagliptin improves glycemic control in insulin-treated Japanese patients with type 2 diabetes mellitus. Endocr J.

[16] Aroda VR, Henry RR, Han J, Huang W, DeYoung MB, Darsow T, Hoogwerf BJ (2012). Efficacy of GLP-1 receptor agonists and DPP-4 inhibitors: meta-analysis and systematic review. Clin Ther.

[17] Rizzo MR, Barbieri M, Marfella R, Paolisso G (2012). Response to Comment on: Rizzo et al. Reduction of oxidative stress and inflammation by blunting daily acute glucose fluctuations in patients with type 2 diabetes: role of dipeptidyl peptidase-IV inhibition. Diabetes Care.

[18] Bando Y, Yamada M, Aoki K, Kanehara H, Hisada A, Okafuji K, Toya D (2014). Predictive clinical parameters for the hemoglobin A1c-loweringeffect of vildagliptin in Japanese patients with type 2 diabetes. Diabetol Int.

[19] Bando Y, Kanehara H, Aoki K, Hisada A, Toya D, Tanaka N (2012). Obesity may attenuate the HbA1c-lowering effect of sitagliptin in Japanese type 2 diabetic patients. J Diabetes Investig.

[20] Esposito K, Chiodini P, Capuano A, Maiorino MI, Bellastella G, Giugliano D (2014). Baseline glycemic parameters predict the hemoglobin A1c response to DPP-4 inhibitors : meta-regression analysis of 78 randomized controlled trials with 20,053 patients. Endocrine.

[21] Iwamoto Y, Taniguchi T, Nonaka K, Okamoto T, Okuyama K, Arjona Ferreira JC, Amatruda J (2010). Dose-ranging efficacy of sitagliptin, a dipeptidyl peptidase-4 inhibitor, in Japanese patients with type 2 diabetes mellitus. Endocr J.

[22] Ishii H, Ohkubo Y, Takei M, Nishio S, Yamazaki M, Kumagai M, Sato Y (2014). Efficacy of combination therapy with sitagliptin and low-dose glimepiride in Japanese patients with type 2 diabetes. J Clin Med Res.

